# Assessment of Immunoscore, MRI Tumor Regression Grade, and Neoadjuvant Rectal Score in Predicting Pathologic Response in Locally Advanced Rectal Cancer in the Averectal Study †

**DOI:** 10.3390/diagnostics15070913

**Published:** 2025-04-02

**Authors:** Mustafa Natout, Ahmad Machmouchi, Hero Hussain, Laudy Chehade, Noura Abbas, Rim Turfa, Joseph Kattan, Sally Temraz, Ayman Tawil, Mousa Elkhaldi, Omar Jaber, Rula Amarin, Tala Alawabdeh, Maya Charafeddine, Monita Al Darazi, Ali Shamseddine

**Affiliations:** 1 Department of Diagnostic Radiology, American University of Beirut Medical Center, Beirut 1107 2020, Lebanon; mn113@aub.edu.lb; 2 Department of Internal Medicine, Division of Hematology/Oncology, Naef K. Basile Cancer Institute—NKBCI, American University of Beirut Medical Center, Beirut 1107 2020, Lebanon; ahmadmach1996@gmail.com (A.M.); lc30@aub.edu.lb (L.C.); na331@aub.edu.lb (N.A.); st29@aub.edu.lb (S.T.); mc16@aub.edu.lb (M.C.); ma99@aub.edu.lb (M.A.D.); 3 Department of Abdominal Radiology, University of Michigan, Ann Arbor, MI 48109, USA; hhussain@med.umich.edu; 4 Department of Internal Medicine, Division of Hematology/Oncology, King Hussein Cancer Center, Amman 11941, Jordan; rturfa@khcc.jo (R.T.); ramarin@khcc.jo (R.A.); ta.11388@khcc.jo (T.A.); 5 Department of Hematology/Oncology, Hotel-Dieu de France University Hospital, Beirut 166830, Lebanon; jkattan62@hotmail.com; 6 Department of Pathology and Laboratory Medicine, American University of Beirut Medical Center, Beirut 1107 2020, Lebanon; at04@aub.edu.lb; 7 Department of Radiation Oncology, King Hussein Cancer Center, Amman 11941, Jordan; mkhaldi@khcc.jo; 8 Department of Pathology, King Hussein Cancer Center, Amman 11941, Jordan; oj.12785@khcc.jo

**Keywords:** immunoscore, rectal cancer, MR tumor regression grade, locally advanced rectal cancer, pathologic complete response

## Abstract

**Background/Objectives**: Predictive tools are needed to assess the response to treatment and guide treatment decisions for locally advanced rectal cancer (LARC). This study explores the value of combining the immunoscore (IS) and magnetic resonance imaging tumor regression grade (mrTRG) with pathologic and radiologic neoadjuvant rectal (NAR) scores in predicting pathologic complete response (pCRs). **Methods**: The scores were assessed for patients with LARC enrolled in the Averectal study (NCT03503630), who received five fractions of short-course radiotherapy, followed by six cycles of mFOLFOX-6 plus avelumab, and total mesorectal excision. The IS was calculated using the mean density percentiles of CD3- and CD8-positive T-cells on baseline biopsy samples. Baseline and post-treatment MRIs were reviewed to measure the mrTRG. NAR scores were calculated using the pre-treatment T stage and post-treatment pathologic and radiologic N and T stages. **Results**: Fifteen out of thirty-five patients whose data were available achieved pCR (42.8%), and seven out of fourteen patients with mrTRG = 1 (complete response) attained pCR. In patients with both a mrTRG = 1 and high IS, the pCR rate was 66.7% (6/9). All of the patients who achieved pCR had a low or intermediate pathologic NAR score with a significant correlation between pCR and pathologic NAR scores (*p* < 0.0001). Both pathologic and radiologic NAR scores were correlated with overall survival and disease-free survival. **Conclusions**: The IS can supplement the mrTRG to better predict TNT outcomes, along with the use of the NAR score. This combination could potentially help with patient selection for non-operative management and guide treatment strategies for those with different recurrence risks.

## 1. Introduction

Colorectal cancer (CRC) is the third most common malignancy in both men and women. In the United States, it represents the third leading cause of new cancer cases and cancer-related deaths. In 2022 alone, 106,180 new cases and 52,580 new deaths were reported. Rectal cancer, which accounts for almost 30% of all CRCs, is now considered one of the most frequent cancers worldwide [1,2].

Over the past few years, the management of rectal cancer has shown remarkable improvement, with an increasing interest in non-operative strategies. This has led to innovations and advances, including organ-preserving strategies for responsive diseases [3,4]. Currently, the standard treatment for locally advanced rectal cancer (LARC) includes a trimodality approach comprising neoadjuvant chemoradiation therapy (nCRT) followed by surgery with total mesorectal excision (TME), and then adjuvant chemotherapy [5,6,7].

However, due to the anatomical location of the rectum, executing such surgeries is particularly challenging. In addition to the operative morbidity, long-term functional sequelae, and impairment of quality of life, surgery for rectal cancer is associated with a higher rate of local recurrence compared to colon cancer [8,9].

Hence, a novel approach has been explored in previous single-arm trials: total neoadjuvant therapy (TNT). TNT is a promising treatment for LARC that attempts to deliver both systemic chemotherapy and nCRT prior to surgery [4,10,11]. Accumulating evidence shows that TNT in patients with LARC is expected to reduce metastasis and improve survival due to high compliance with chemotherapy and subsequently higher pathological complete response (pCR) rates [12]. TNT has also been associated with decreased toxicity and a reduced need for ileostomy [13,14,15,16,17,18].

pCR after nCRT for rectal cancer is a predictor of both decreased local relapse and improved disease-free survival (DFS) [19,20,21]. Yet, no reliable indicators are capable of accurately predicting pCR before surgery; as a result, the use of a specific prognostic tool is still needed. Therefore, it is crucial to determine predictive factors for pCR in order to select patients eligible for non-operative management (NOM) or the watch-and-wait (W&W) approach [22,23].

Recently, the use of MRI (MR tumor regression grade; mrTRG) has been implemented as a prognostic tool to provide information and evaluate the response of rectal cancer to nCRT, pathological response, and cancer-specific prognosis. Therefore, it might be a promising guide for these treatments [5]. However, its ability to predict pCR needs to be improved [24]. Wang et al. [24] reported that 46.6% of patients with magnetic resonance tumor regression grade 1 (mrTRG = 1; complete radiologic response) achieved pCR (no residual tumor cells).

The management of LARC continues to evolve, as several clinical trials evaluating different treatment approaches are emerging. However, clinically relevant treatment results, such as improvement in survival, require time to be validated, usually between 3 and 5 years, which can delay the application of new therapies into clinical practice. To this end, the neoadjuvant rectal (NAR) score was proposed as a surrogate endpoint for survival in clinical trials. It was conceptualized using variables associated with treatment effect, based on the Valentini nomogram that predicts the response to TNT in LARC. The NAR score is calculated using the pathologic T and N stages and the initial clinical T stage to account for tumor regression even if a complete pathologic response is not reached [25]. Even though this score was validated in the NSABP R-04 trial that included 1479 patients and was found to strongly correlate with overall survival (OS), outperforming pCR, the evidence from other studies in the literature is conflicting. Furthermore, the utility and generalizability of the NAR score are based on trials involving long-course chemoradiotherapy (LCCRT), and to our knowledge, it has not been used with TNT regimens involving short-course radiotherapy (SCRT) or immunotherapy, apart from one trial, the NRG-GI002 [26]. A recent imaging sub-study of this trial examined the ability of MRI to predict pCR and found that mrTRG significantly correlated with pCR and NAR scores [27]. The NAR score is categorized into low, intermediate, or high, with low scores correlating with better OS and DFS [25].

We previously reported, in the Averectal study, the use of the immunoscore (IS) as a validated prognostic tool to predict pCR. Results revealed a significant association between high pretreatment IS and pCR, with 15 (42.8%) patients achieving a mean IS of 68 (±22 SD) in patients with a high IS as opposed to 52 (±22 SD) in patients with a low IS (*p* = 0.036) [28]. In this prospective cohort study, we explore the value of the IS in combination with mrTRG in the prediction of pCR before surgery and investigated the utility of the IS in risk stratification and prognostic prediction for patients undergoing nCRT and TME. In addition, we explore the relationship of pathologic and radiologic versions of the NAR score with pCR and survival in our cohort.

The primary aim of this study is to assess the use of a combination of the mrTRG, IS, and NAR score in predicting pCR in patients with LARC who received nCRT as part of TNT in the Averectal phase II study. The secondary aims include evaluating the correlation between the IS and mrTRG in predicting DFS and OS and assessing the correlation between the NAR score and DFS and OS.

## 2. Materials and Methods

### 2.1. Study Design

The Averectal study (NCT03503630; date of registration 29 March 2018) is a principal investigator-initiated, open-label, multicenter, prospective phase II study. This study investigates the efficacy and safety of 5 fractions of short-course radiotherapy, followed by 6 cycles of mFOLFOX-6 plus avelumab, followed by TME, in patients with LARC (cT2 N1−3, cT3 N0−3, and cT4a N0−3 evidence of extramural vascular or mesorectal fascia involvement), with a tumor located less than 15 cm from the anal verge. Exclusion criteria included distant metastasis, cT2N0 or cT4b disease, recurrent rectal cancer, prior radiotherapy or chemotherapy, symptoms of peripheral neuropathy, current immunosuppressive medication, active autoimmune disease, active infection, vaccination within 4 weeks of the first dose of avelumab, history of human immunodeficiency virus or acquired immunodeficiency syndrome, or hepatitis B or hepatitis C viral infection at screening. The study was approved by the local Institutional Review Board (IRB) Committee at the American University of Beirut Medical Center (AUBMC), Hotel Dieu de France, Beirut, and King Hussein Cancer Center, Amman, in accordance with ethical guidelines for biomedical research.

**Figure 1:**
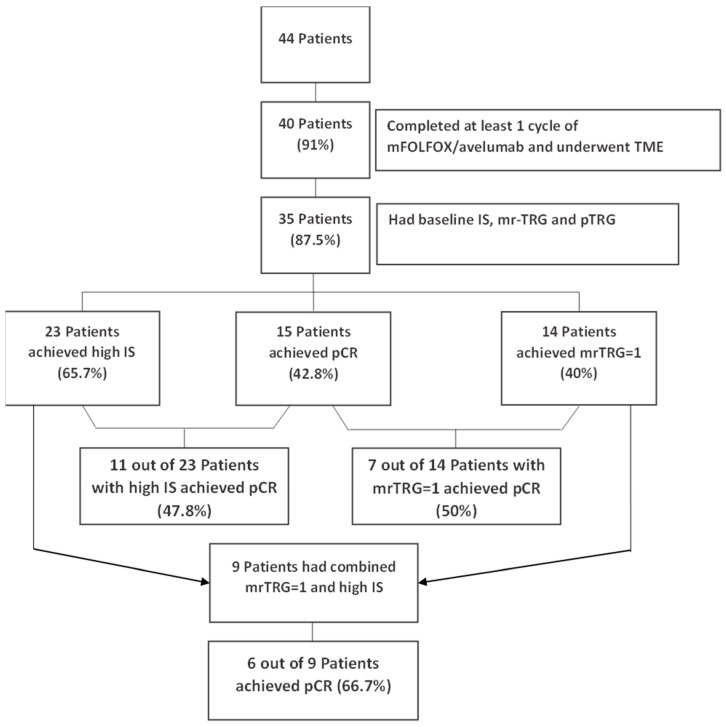
Study design and results.

All patients provided written informed consent before their enrollment in the study. Data from the medical records of 44 patients who were pathologically diagnosed with LARC and enrolled in the Averectal study between 2018 and 2020 were used (Figure 1). The data included patient demographics, anatomic locations, grade, stage, and outcome of the disease.

### 2.2. Tumor Characteristics

We relied on pathology reports obtained either by surgical resection or tissue biopsy performed at our center in order to identify the patients’ profiles. These reports included several tumor features such as size, location, size, invasion, stage, and grade. Rectal cancer TNM staging was performed using the American Joint Committee on Cancer (AJCC) Union for International Cancer Control (UICC) 8th edition.

### 2.3. Rectal MRI Methodology

The pre- and post-treatment rectal MRIs of 35 patients were available and assessed using the same systemic approach. Patients were imaged on high-field MRIs (3 Tesla) in the supine position with a pelvic phased-array surface coil.

The MRI protocol included a large field of view (FOV) localizer, followed by triplanar high-resolution two-dimensional (2D) T2-weighted Turbo Spin Echo (TSE) sequences, with sagittal T2-TSE oriented along the axis of the pelvis for visualization of the rectal mass, and oblique axial and oblique coronal high-resolution small FOV T2-TSE oriented along the short and long axis of the rectal mass, respectively. The protocol included oblique axial diffusion-weighted echoplanar imaging (DWI) with increasing *b* values (*b* = 50, 600, 1000 s/mm^2^) and generated ADC maps.

Additional optional sequences included fat saturation axial T2-weighted sequences and three-dimensional (3D) volumetric interpolated T1-weighted gradient echo (GRE) (axial non-enhanced followed by axial, coronal, and sagittal contrast-enhanced) sequences following the intravenous administration of gadolinium-based contrast agent such as Dotarem^®^ (gadoterate meglumine), 0.2 mL/kg.

On T2-weighted FSE sequences, non-mucinous rectal tumors appear as intermediate signal lesions on a background of low-signal-intensity muscularis propria, while mucinous tumors appear as high-signal-intensity lesions [2].

The primary tumor (T) and its extension (depth of invasion into surrounding mesorectal fat) were assessed on the high-resolution T2-weighted TSE sequences (depicting rectal wall layers’ involvement). No T1 and only one T2 tumor was encountered (limited to rectal mucosa and muscularis propria, respectively). The rest of the tumors were T3 tumors (extending beyond the muscularis propria into the mesorectal fat) and were subclassified into T3a (<1 mm depth of invasion), T3b (1–5 mm), T3c (5–15 mm), and T3d (>15 mm) [29]. No T4a tumors were encountered (tumors invading peritoneal reflections).

High-resolution T2-weighted TSE sequences were also used to evaluate the distance from and involvement of the mesorectal fascia (MRF), mesorectal fat, and peritoneal reflections on the oblique axial T2 sequence. The distance of the tumor from the anal verge and the top of the anal canal, as well as from the peritoneal reflections, was evaluated on the sagittal T2 sequence. The coronal T2 sequence was used to assess the involvement of the anal sphincter complex (internal and external sphincters and intersphincteric space) [2].

DWI was included in all studies as it improves primary tumor as well as metastatic lymph node detection in primary staging [2].

Following identification of the rectal mass, the systemic approach included assessment of the following items on the pre- and post-treatment rectal MRIs:

- Craniocaudal length of the tumor and short-dimension thickness of the mass;
- Distance of the tumor from the anal verge and from the top of the anal sphincter complex;
- Mucinous nature of the tumor;
- Extramural depth of invasion;
- Relationship to peritoneal reflections;
- Invasion of peritoneal reflections;
- Invasion of adjacent organs;
- Invasion of anal canal;
- Number of visible mesorectal lymph nodes and size of largest lymph node;
- Minimum distance of primary tumor/metastatic lymph node/mesorectal deposit to mesorectal fascia;
- Presence of extramural vascular invasion (EMVI);
- Presence of mesorectal fascia invasion (status of the circumferential resection margin (CRM)).

Rectal tumors were categorized depending on the distance of the inferior edge of the mass from the anal verge, as low (0–5 cm from the anal verge), middle (5–10 cm), and high (10–15 cm). Circumferential tumoral involvement of the rectum was assessed in the clockface position.

Nodal assessment relied primarily on size (6 mm short dimension being cut-off for suspicious metastatic lymph nodes), signal intensity (whether similar to the primary tumor), and irregular contour. Extramural vascular invasion, if present, appeared as thickening of small vessels adjacent to the primary tumor, showing irregular lobulated appearance and signal intensity similar to the primary tumor as well as heterogeneous enhancement post-contrast administration.

**Figure 2:**
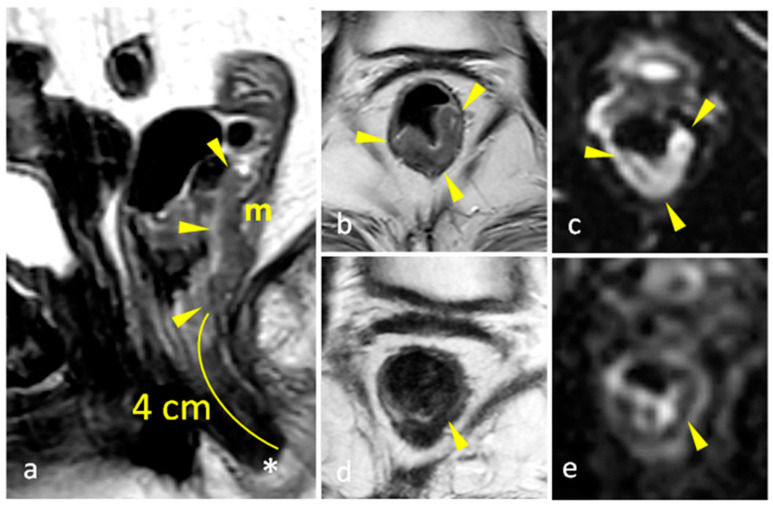
Pretreatment (**a**) sagittal T2-weighted TSE showing low rectal mass (m), seen 4 cm from the anal verge (*) extending for a craniocaudal length of 3.5 cm (between arrowheads) and showing intermediate signal intensity. (**b**) Axial T2-weighted TSE showing the rectal mass (arrowheads), involving the posterior and left lateral rectal walls, and extending from 1 till 8 o’clock in a clockwise direction. (**c**) Axial DWI of the same mass showing restricted diffusion. Post-treatment (**d**) axial T2-weighted TSE and (**e**) axial DWI at the same level, showing normal rectal wall and suggesting complete radiologic response (mrTRG 1).

**Figure 3:**
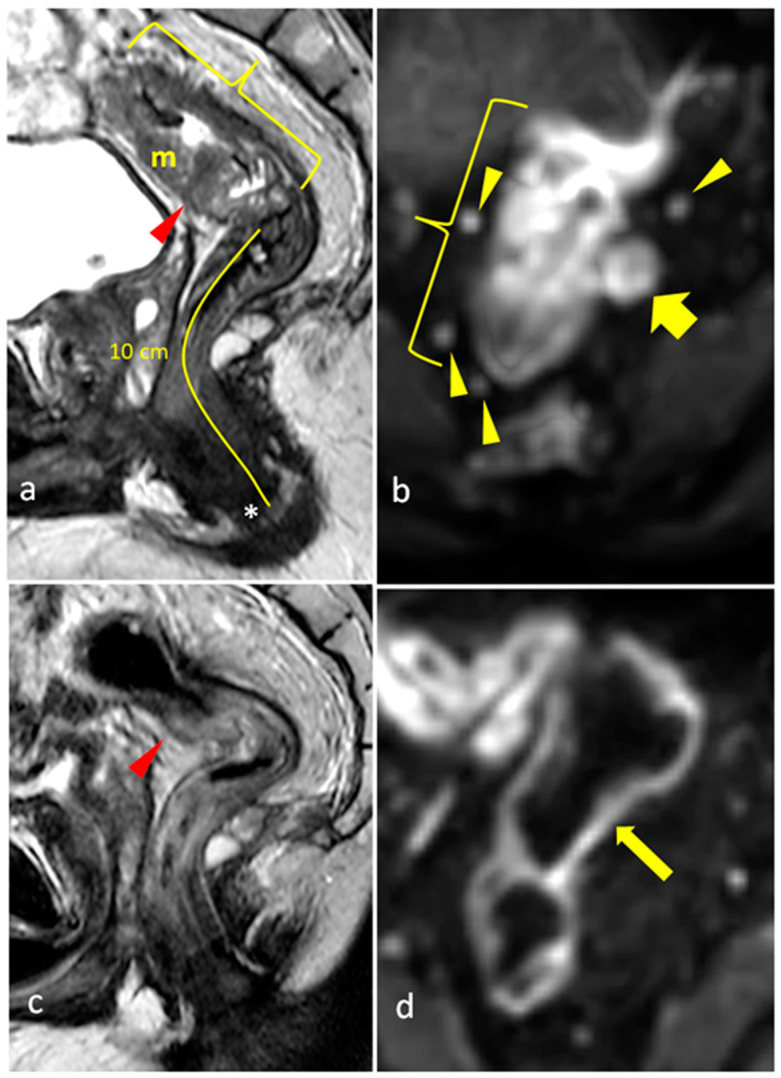
Pretreatment (**a**) sagittal T2-weighted TSE showing intermediate signal intensity and high rectal mass (m), seen at 10 cm from the anal verge (*) extending for a craniocaudal distance of 8 cm (bracket) and encroaching on the peritoneal reflection (red arrowhead). (**b**) Coronal DWI of the same mass showing diffusion restriction, associated with several mesorectal lymph nodes (arrowheads) and a mesorectal metastatic deposit (short arrow). Post-treatment (**c**) sagittal T2-weighted TSE showing complete radiologic response with rectal wall edema at the site of the mass (thickened rectal wall with high signal intensity), and corresponding (**d**) DWI showing resolution of the mass and metastatic deposit (mrTRG 1).

**Figure 4:**
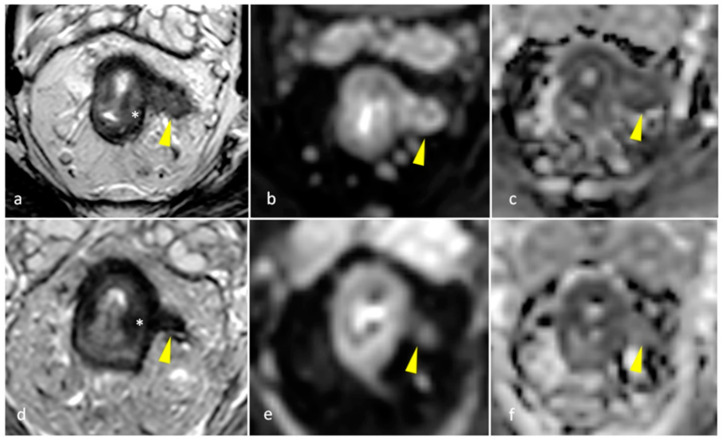
Pretreatment (**a**) axial T2-weighted TSE showing EMVI (arrow) along the left aspect of a rectal mass (*) as an intermediate-signal-intensity lobulated, elongated structure showing high signal on (**b**) DWI and restricted diffusion (low signal) on (**c**) ADC map. Post-treatment images show decreased signal of the rectal mass (*) and thinning of EMVI (arrow), appearing as a low signal intensity fibrotic band on (**d**) T2-weighted TSE, with a faint high-signal-intensity structure on (**e**) DWI, with no restricted diffusion (high signal) on (**f**) ADC map, suggesting replacement of tumoral tissue with fibrosis (mrTRG 2).

The status of the CRM was assessed by measuring the shortest distance between the outermost part of the rectal tumor and the MRF [2]. Expected post-treatment changes included a decrease in the size of the rectal tumors, a decrease in signal intensity (fibrotic changes) or an increase in the signal intensity of non-mucinous masses (mucin deposition/colloidal degeneration), a decrease in the tumoral depth of invasion, a decrease in size and number of visible mesorectal and pelvic lymph nodes, and a decrease/resolution of EMVI (low T2 signal in involved vessels or resolution of the irregular thickening) (as shown in Figure 2, Figure 3 and Figure 4). Such changes lowered the TNM staging from T3 to T0 in many patients. The rest of the items of the systemic approach were also re-assessed, including the distance from and involvement of the anal sphincter complex. High-resolution T2 TSE and DWI were reviewed to assign the mrTRG, and fibrotic changes were additionally evaluated on post-contrast 3D-GRE T1-weighted sequences.

### 2.4. Immunohistochemical Methodology

Immunohistochemical analysis was performed on baseline biopsies using formalin-fixed, paraffin-embedded tissue sections of thickness 5 µm. The deparaffinization and immunohistochemical staining were carried out using a microwave streptavidin immunoperoxidase (MSIP) protocol and labeled streptavidin–biotin (LSAB) method on a DAKO TechMate™ Horizon automated immunostainer (Agilent Technologies, Santa Clara, CA, USA).

Immunohistochemical staining for CD3 and CD8 was performed to identify and quantify T-cell densities, enabling the calculation of the IS. Pathology reviews were conducted using surgical pathology blocks to evaluate tumor regression grades and immune cell infiltration. Regions of interest for analysis were selected based on proximity between tumor cells and surrounding normal tissue, ensuring representative sampling. Areas with the highest density of infiltrating immune cells were prioritized for cell counting to enhance accuracy.

**Figure 5:**
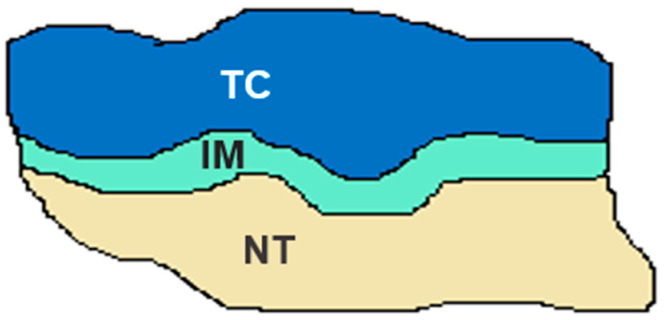
Representation of IS definition (CD3^+^ and CD8^+^ T-cell density in the TC and IM). IM, invasive margin; NT, normal tissue; TC, tumor core.

Two specific regions were targeted for evaluation: the invasive margin (IM), defined as the interface between the tumor and the adjacent normal tissue, and the core of the tumor (CT), including the tumor stroma and tumor glands (Figure 5). Tumor-infiltrating lymphocyte (TIL) densities were assessed under a microscope at 40× magnification, focusing on the richest areas of stained cells within both the IM and CT regions, regardless of tissue sample size. Counts of CD3- and CD8-positive T-cells were performed separately for each region. The IS was calculated as the mean of four recorded values: CD3 (CT), CD3 (IM), CD8 (CT), and CD8 (IM). This standardized approach ensured a detailed assessment of immune cell distribution and density. To classify the IS, a cutoff value of 62% was applied to distinguish between high- and low IS-groups (Table 1).

### 2.5. Statistical Analysis

Numerical variables were summarized by their median, mean +/− SD, and range. Categorical variables were described by counts and relative frequencies. Mean comparison was performed using an independent sample *t*-test for the proportion of high-IS patients who achieved pCR versus patients with an mrTRG who attained pCR, and versus patients with a combined high IS and mrTRG who achieved pCR.

The pathologic NAR score was calculated based on the following formula [27]: $\frac{{\left[5\;pN-3\left(cT-pT\right)+12\right]}^{2}}{9.61}$, where *pT* and *pN* indicate the post-treatment pathologic *T* and *N* stages, and *cT* is the baseline clinical *T* stage of MRI.

A radiologic version of the NAR score (radiologic NAR) was calculated as follows [27]: $\frac{{\left[5\;{cN}_{post\;TNT}-3\left({cT}_{pre\;TNT}-{cT}_{post\;TNT}\right)\right]}^{2}}{9.61}$, where *cT_preTNT_* is the pretreatment *T*, and *cN_postTNT_* and *cT_postTNT_* are the *N* and *T* stages of post-treatment MRI.

The NAR scores were categorized according to their value into low (less than 8), intermediate (between 8 and 16), and high (more than 16). This categorization is based on previous studies from the literature (Table 1).

Crosstabs were tabulated in 2 × 2 tables comparing pCR with pathologic NAR and pCR with radiologic NAR scores.

The DFS time was defined as the time from initial diagnosis to disease progression or the end of follow-up (censored observations that did not reach the progression event). The DFS curve was plotted using the Kaplan–Meier method, and the log-rank test was used to check for significant differences between the studied groups.

All *p* values were 2-sided. A value of *p* < 0.05 was considered significant in all analyses. All statistical analyses were performed using the SPSS v.25.0 statistical package.

## 3. Results

**Figure 6:**
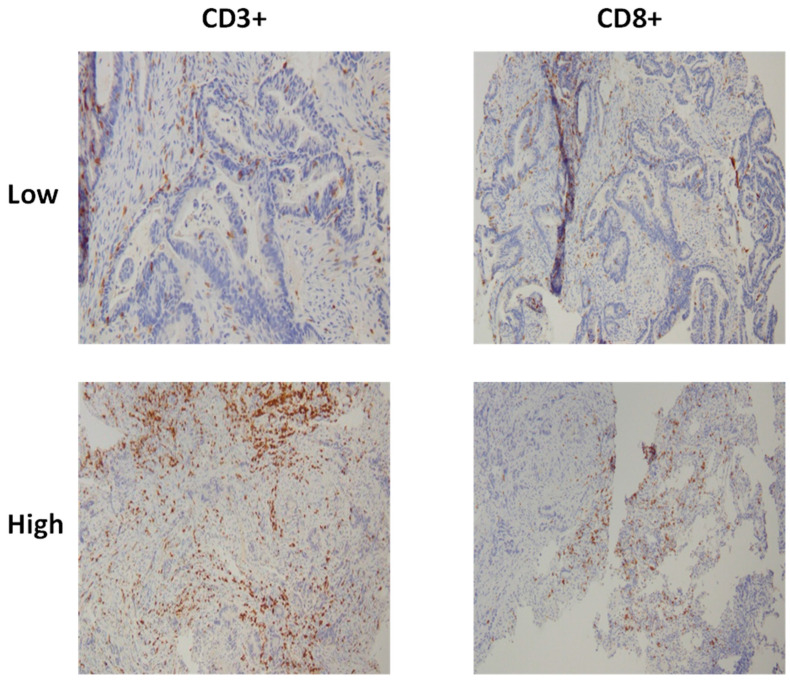
IS of 2 locally advanced rectal cancer samples after CD3^+^ and CD8^+^ T-cell immunostaining.

Between July 2018 and October 2020, 44 patients were accrued, out of which 40 (90%) completed at least one cycle of mFOLFOX/avelumab and underwent TME. Of these 40 patients, 35 (87.5%) had their baseline IS (Figure 6), mrTRG (post-treatment), and pathologic tumor regression grade (pTRG) assessed. Out of these 35 patients, 15 (42.8%) achieved pCR, 14 (40%) had a mrTRG = 1, and 23 (65.7%) had a high IS.

**Figure 7:**
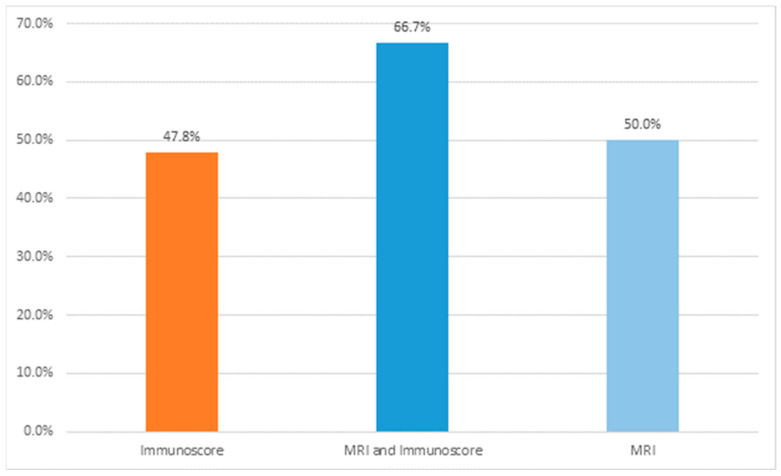
Proportion of pCR for high IS and/or high MRI TRG.

Moreover, seven out of the fourteen patients (50.0%) with a mrTRG = 1 attained pCR, whereas 11 out of 23 patients (47.8%) with a high IS achieved pCR (Figure 7). Most importantly, of the patients with a combined high IS and mrTRG = 1 (*n* = 9), six (66.7%) achieved pCR (Table 2). These results are different from the pCR rate for patients with either a high IS (11/23, 47.8%) or mrTRG = 1 (7/14, 50%), as shown in Figure 1 and Figure 7. Most patients (32/35) had moderately differentiated tumors (grade 2). Among patients who achieved pCR, twelve (92.31%) were grade 2 and one (7.69%) was grade 3; among patients who did not achieve pCR, one (4.45%) was grade 1, twenty (90.90%) were grade 2, and one (4.45%) was grade 3.

**Figure 8:**
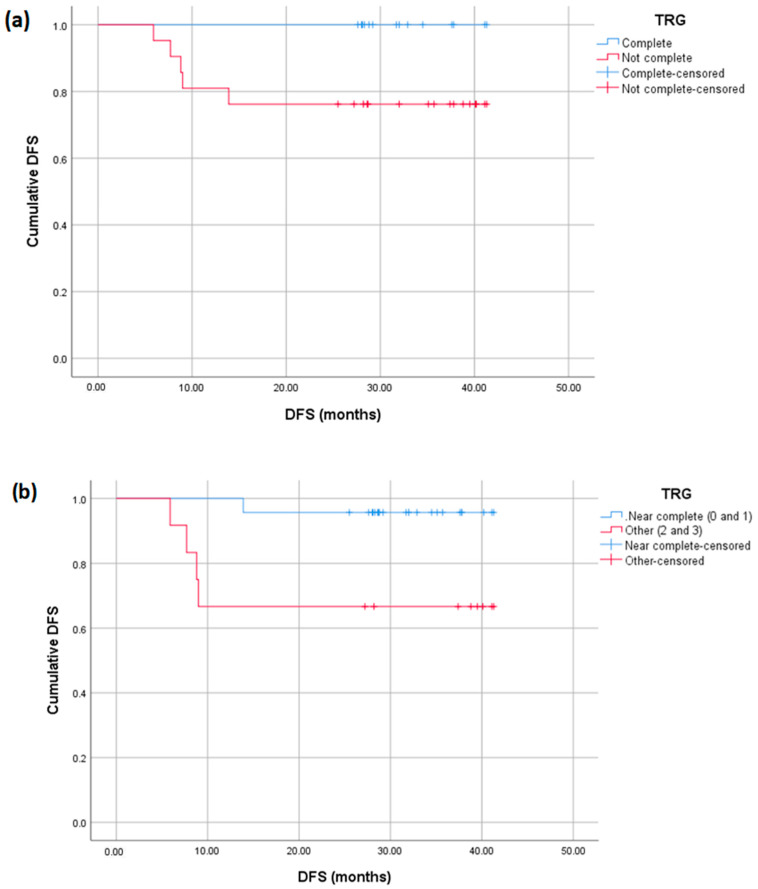
Kaplan–Meier curves displaying the DFS of patients with and without TRG 0 (**a**) and with or without near pCR (**b**).

Finally, as shown in Figure 8, both pCR and near pathologic complete response (pTRG ≤ 1) significantly correlate with DFS (*p* = 0.05 and *p* = 0.015), respectively. When assessing OS by the IS level and mrTRG = 1 at 3 years, our results showed that patients with a high IS and mrTRG = 1 had 100% survival compared to 92% in patients with the rest of the population.

All of the patients who achieved pCR had a low or intermediate pathologic NAR score. Specifically, 92.3% of patients who achieved pCR had a low pathologic NAR score, while only 7.7% of patients with an intermediate NAR score achieved pCR, and none with a high pathologic NAR score achieved pCR. Among the patients who did not achieve pCR, 18.2% had a low NAR score, 40.9% had an intermediate score, and 40.9% had a high score. Fisher’s exact test showed a significant correlation between pCR and NAR score (*p* < 0.0001). All the patients who achieved pCR had a low or intermediate radiologic NAR score, but the correlation between radiologic NAR and pCR was not statistically significant (*p* = 0.128), as presented in Table 2.

**Figure 9:**
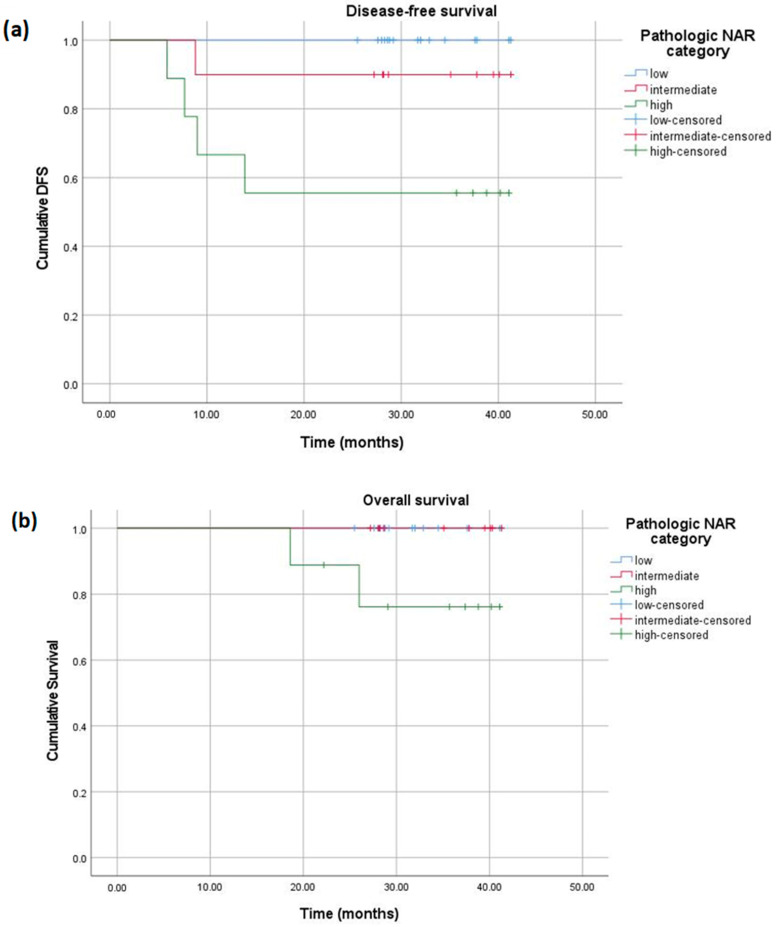
Kaplan–Meier curves displaying the DFS (**a**) and OS (**b**) of patients with low (blue), intermediate (red), and high (green) pathologic NAR scores.

**Figure 10:**
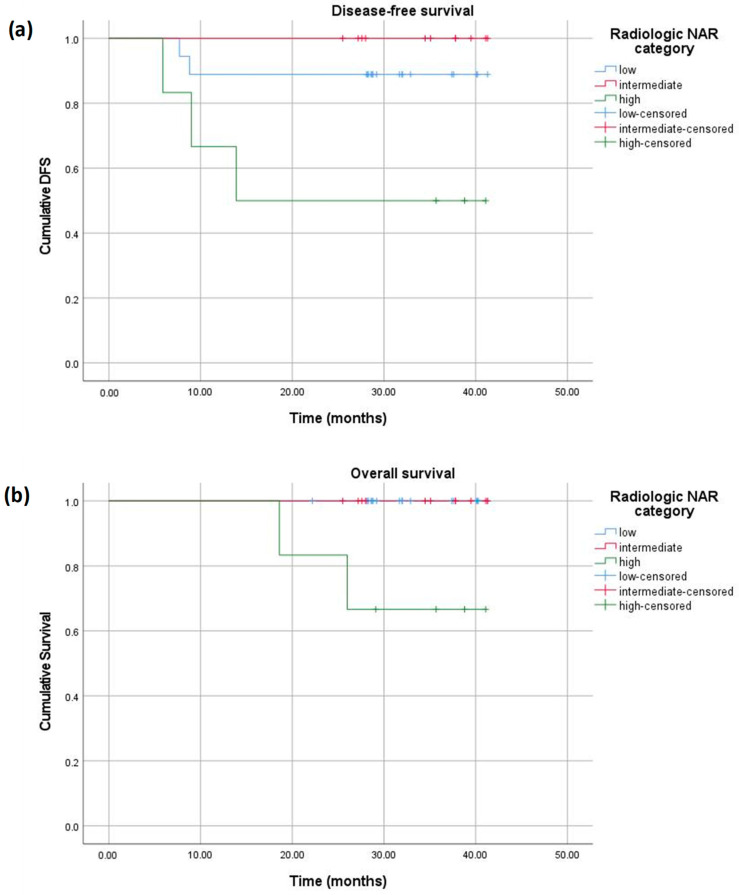
Kaplan–Meier curves displaying the DFS (**a**) and OS (**b**) of patients with low (blue), intermediate (red), and high (green) radiologic NAR scores.

At the end of the 40-month follow-up, both pathologic and radiologic NAR scores correlated with OS and DFS. DFS was 100% for patients with a low pathologic NAR, 90% for those with an intermediate score, and 55.6% for those with a high score (*p* = 0.007). OS was 100% for patients with low and intermediate pathologic NAR scores and 77.8% for those with a high pathologic NAR score (*p* = 0.04) (Figure 9). When stratified according to the radiologic NAR score, patients with a low NAR had no disease progression and no death, those with an intermediate score had a DFS of 90% and OS of 100%, and finally, those with a high NAR score had a DFS of 50% and OS of 65% (Figure 10).

## 4. Discussion

The results of our study demonstrate that SCRT followed by mFOLFOX/avelumab combination therapy can lead to a high rate of tumor regression in patients with LARC. Our findings validate that both the baseline IS and mrTRG are important predictors of treatment response. Notably, patients with both a high IS and mrTRG = 1 had the highest rate of pCR, at 66.7%. This significant finding highlights the importance of combining these two tools to identify patients who may benefit most from the NOM approach.

A particular study conducted by Maas et al. [30] evaluated the long-term results of rectal cancer patients following nCRT, using MRI and endoscopy plus biopsies. Out of 21 patients who had a clinical complete response and were included in the W&W approach, only one developed a local recurrence and had surgery as salvage treatment. The control group, consisting of 20 patients achieving pCR after surgery, showed two-year DFS and OS rates of 93% and 91%, respectively.

In a systemic review and meta-analysis, Jang et al. [31] assessed the diagnostic accuracy of mrTRG for pCR by combining six studies, including 916 patients. The meta-analytic summary sensitivity and specificity of a mrTRG = 1 for pCR were 32.3% and 93.5%, respectively. Wang et al. reported that 46.6% of patients with a mrTRG = 1 achieved pCR [24,32]. These results revealed that a NOM approach, with strict selection criteria and adequate follow-up using up-to-date imaging techniques (MRI), led to promising outcomes [33].

In our open-label, phase II study, 14 out of 35 patients (40%) were reported to have a mrTRG = 1. Moreover, 7 out of the 14 patients (50%) attained pCR, revealing results consistent with the literature. However, the question remains whether we can supplement MRI with another prognostic tool to better predict nCRT response, potentially improving patient selection for NOM and guiding the treatment strategy.

The prognostic significance of the IS for LARC was demonstrated in various studies; the 5-year survival rate was higher in patients with high a IS than with low IS [34]. Additionally, in many solid tumors, strong immune infiltration of the tumor has been associated with prolonged survival [35,36]. Specifically, a high infiltration of CD8^+^, CD4^+^, and CD3^+^ T lymphocytes localized in the margin and the core of the tumor has been correlated with a prolonged time in therapeutic range, increased OS, and DFS [37,38,39,40].

In a study conducted by El Sissy et al. [41], biopsies from two independent cohorts (n1 = 131, n2 = 118) were taken from patients with LARC, treated with nCRT followed by radical surgery, and were immunostained for CD3^+^ and CD8^+^ T-cells and quantified by digital pathology to determine the IS. Results were correlated with responses to nCRT and DFS. The IS prognostic performance was further assessed in a multicentric cohort (*n* = 73 patients) treated by the W&W approach. The IS was positively correlated with the degree of histologic response (*p* < 0.001) and gene expression levels for Th1 orientation and cytotoxic immune response, post-nT (*p* = 0.006). A high IS identified patients at lower risk of relapse or death compared to a low IS. In the W&W cohort (*n* = 73), no relapse was observed in patients with a high IS (23.3%) [41].

Similar results were showcased in our phase II Averectal study, establishing the use of the IS as a validated prognostic tool in LARC. Our results revealed that 15 (41.6%) patients with pCR had a mean IS of 68 (±22 SD) in patients with a high IS, compared to 52 (±22 SD) in patients with a low IS (*p* = 0.036) [28]. Consistent with these data, rectal tumors with high numbers of infiltrating CD3^+^ and CD8^+^ T-cells have higher response rates to nCRT [42].

A meta-analysis of 15 trials found that TNT appears to have advantages over standard therapy for LARC in terms of pCR, R0 resection, DFS, and OS, with comparable nCRT [43]. The pCR rate was significantly higher in the TNT group than in the nCRT group (OR 1.85, 95% CI 1.39–2.46, *p* < 0.0001), with higher DFS (HR 0.80, 95% CI 0.69–0.92, *p* = 0.001), and OS (HR 0.75, 95% CI 0.62–0.89, *p* = 0.002). Our study yielded comparable results, with pCR and near pCR significantly correlated with DFS (*p* = 0.05 and *p* = 0.015, respectively). The significant difference further highlights the role of pCR as a valuable predictor of favorable survival outcomes.

The NAR score was developed as a short-term surrogate endpoint for survival in clinical trials concerning the neoadjuvant treatment of LARC. This score reflects the effects of treatment on the T stage, accounting for tumor regression even if it is not to the point of pCR [25]. The NAR score was validated in the NSABP R-04 trial, in which patients with stage II or III rectal cancer were randomized to receive one of the following neoadjuvant CRT radio-sensitizers: 5-FU, 5-FU with oxaliplatin, capecitabine, or capecitabine with oxaliplatin. The three score categories (low, intermediate, and high) were significantly correlated with 5-year OS, which was 92%, 89%, and 68%, respectively (*p* < 0.0001) [44]. Our study, which used a different neoadjuvant regimen consisting of SCRT and chemo-immunotherapy, showed similar results. The NAR score was found in several studies to better correlate with OS than pCR. In the Averectal trial, the pathologic NAR score significantly correlated with pCR, as patients with lower scores tended to have a higher percentage of pCR. The score categories were also significantly correlated with DFS, as we reported above, which was also corroborated by Hong et al. [45] and Roy et al. [46], and in the CAO/ARO/AIO-04 later on [47].

The efficacy of SCRT followed by mFOLFOX-avelumab combination therapy and total mesorectal excision (TME) was evaluated in the Averectal study [48], which demonstrated a numerically higher pCR rate compared to the RAPIDO trial (37.5% vs. 28%), despite fewer cycles of chemotherapy (6 vs. 9 cycles). While avelumab is not currently approved for colorectal cancer and is primarily indicated for advanced metastatic cases, its inclusion in this study was based on evidence suggesting that combining immunotherapy with chemotherapy and radiotherapy may yield synergistic effects, enhancing treatment efficacy. However, further phase III trials are required to confirm the role of avelumab in this setting.

Furthermore, in a recently published sub-analysis of the NSABP R-04 trial, the mrTRG was correlated with the magnitude of pathologic response, represented by the NAR score. The authors also propose a radiologic NAR score, which takes into account pre- and post-treatment tumor T stages, which was found to be correlated with the pathologic NAR score [27]. The radiologic NAR score was found to be correlated with OS and DFS but did not significantly predict pCR in our study. This limitation may be explained by the reliance of radiologic NAR scores on imaging techniques that cannot always capture the true extent of tumor regression, particularly when residual microscopic disease is present. Variations in MRI interpretation and subjectivity in assessing tumor response introduce variability to the radiologic NAR score. Furthermore, post-treatment changes, such as fibrosis and inflammation, complicate differentiation between viable tumor tissue and treatment effects. These challenges highlight the need for further refinement and validation of the radiologic NAR score as a predictive tool for pCR, which may be useful in selecting candidates for NOM of LARC after neoadjuvant treatment.

The results of the phase II Averectal Study provide important insights into the use of the IS, mrTRG, and NAR scores to predict complete pCR in patients with LARC. However, this study was limited by the small sample size (35 patients), with only nine patients having high a IS and mrTRG = 1, which may impact the generalizability and robustness of our results. Additionally, the correlation between these pathologic parameters may be influenced by confounding factors, such as variations in tumor biology, immune microenvironment, and technical variations in imaging and immunohistochemical analysis. Despite these limitations, our findings highlight the potential clinical utility of these biomarkers in guiding personalized treatment strategies. Specifically, their predictive value for pCR could support NOM approaches and help stratify patients based on recurrence risk, allowing for tailored treatment plans that improve patient outcomes. Larger studies are needed to validate these findings and translate their application into clinical practice.

## 5. Conclusions

The model combining the IS and mrTRG improved the predictive performance compared with models derived from individual information. The pathologic and radiologic NAR scores were both correlated with OS and DFS, but only the pathologic NAR score showed a significant correlation with pCR. Therefore, the IS can supplement the mrTRG to better predict TNT outcomes, as well as the use of the NAR score. This combination could potentially help patient selection for NOM and guide the treatment strategy for those with different recurrence risks.

## Figures and Tables

**Table 1:** Criteria for classifying mrTRG, pTRG, IS, and NAR score.

	Grade	Definition
mrTRG	1	Complete Radiologic Response (i.e., no evidence of tumor)
	2	Good Response (i.e., dense >75% fibrosis with no obvious residual tumor)
	3	Moderate Response (i.e., >50% fibrosis or mucin with a minority of visible tumor)
	4	Slight Regression (i.e., <50% fibrosis or mucin with a majority of visible tumor)
	5	No post-treatment changes
pTRG	0	No residual tumor cells
	1	<10% residual tumor cells
	2	10% to 50% residual tumor cells
	3	>50% residual tumor cells
IS	0	>62% mean density percentiles of CD3- and CD8-positive T-cells
	1	<62% mean density percentiles of CD3- and CD8-positive T-cells
NAR	Low	<8
	Intermediate	8 to 16
	High	>16

**Table 2:** Correlation of pCR with pathologic and radiologic NAR scores.

Pathologic NAR	Low, *n* (%)	Intermediate, *n* (%)	High, *n* (%)	*p*-Value
Non-pCR	4 (18.2%)	9 (40.9%)	9 (40.9%)	<0.0001
pCR	12 (92.3%)	1 (7.7%)	0 (0%)	
Radiologic NAR	Low	Intermediate	High	*p*-value
Non-pCR	10 (45.5%)	6 (27.3%)	6 (27.3%)	0.128
pCR	8 (61.5%)	5 (38.5%)	0 (0%)	

## Data Availability

The data supporting the findings of this study are available upon request from the corresponding author.

## Abbreviations

2D, Two-dimensional; 3D, Three-dimensional; AUBMC, American University of Beirut Medical Center; AJCC, American Joint Committee on Cancer; CRC, Colorectal cancer; CRM, Circumferential resection margin; DFS, Disease-free survival; DWI, Diffusion-weighted echo-planar imaging; EMVI, Extramural vascular invasion; FOV, Field of view; GRE, Gradient echo; IRB, Institutional Review Board; IS, Immunoscore; LARC, Locally advanced rectal cancer; LCCRT, Long-course chemoradiotherapy; LSAB, Labeled streptavidin-biotin; MRF, Mesorectal fascia; mrTRG, Magnetic resonance imaging tumor regression grade; MSIP, Microwave streptavidin immunoperoxidase; NAR, Neoadjuvant rectal; nCRT, neoadjuvant chemoradiation therapy; NOM, Non-operative management; OS, Overall survival; pCR, Pathologic complete response; SCRT, Short-course radiotherapy; T, Tumor; TME, Total mesorectal excision; TNT, Total neoadjuvant therapy; TSE, Turbo spin echo; UICC, Union for International Cancer Control; W&W, Watch-and-wait.
